# Inherent transcriptional signatures of NK cells are associated with response to IFNα + rivabirin therapy in patients with Hepatitis C Virus

**DOI:** 10.1186/s12967-015-0428-x

**Published:** 2015-03-01

**Authors:** Maria Libera Ascierto, Federica Bozzano, Davide Bedognetti, Francesco Marras, Cathy Schechterly, Kentaro Matsuura, Antonino Picciotto, Simona Marenco, Yingdong Zhao, Valeria DeGiorgi, Michele Sommariva, Lorenzo Moretta, Ena Wang, Harvey J Alter, Francesco M Marincola, Andrea De Maria

**Affiliations:** Department of Health Sciences, University of Genova, Genova, Italy; Department of Oncology, Johns Hopkins Medicine, CRB II, Room 506, 1550 Orleans Street, Baltimore, MD 21231 USA; Department of Experimental Medicine, University of Genova, Genova, Italy; Sidra Medical and Research Centre, Doha, Qatar; Istituto Giannina Gaslin, Genoa, Italy; Department of Transfusion Medicine, Clinical, National Institutes of Health, Bethesda, MD USA; IRCCS Az.Osp.Univ. San Martino-IST Istituto Nazionale Ricerca sul Cancro, Genoa, Italy; Biometric Research Branch, Division of Cancer Treatment and Diagnosis, National Cancer Institute, National Institutes of Health, Bethesda, MD USA; Universita’ degli Studi di Genova, Largo R Benzi 10, 16132 Genova, Italy

**Keywords:** NK cells, HCV infection, Clinical response, Regulation of NK cells

## Abstract

**Background:**

Differences in the expression of Natural Killer cell receptors have been reported to reflect divergent clinical courses in patients with chronic infections or tumors. However, extensive molecular characterization at the transcriptional level to support this view is lacking. The aim of this work was to characterize baseline differences in purified NK cell transcriptional activity stratified by response to treatment with *PEG*-*IFN*α*/RBV* in patients chronically infected with HCV.

**Methods:**

To this end we here studied by flow cytometer and gene expression profile, phenotypic and transcriptional characteristics of purified NK cells in patients chronically infected with HCV genotype-1 virus who were subsequently treated with *PEG*-*IFN*α*/RBV.* Results were further correlated with divergent clinical response obtained after treatment.

**Results:**

The pre-treatment transcriptional patterns of purified NK cells from patients subsequently undergoing a sustained virologic response (SVR) clearly segregated from those of non-responder (NR) patients. A set of 476 transcripts, including molecules involved in RNA processing, ubiquitination pathways as well as HLA class II signalling were differently expressed among divergent patients. In addition, treatment outcome was associated with differences in surface expression of NKp30 and NKG2D. A complex relationship was observed that suggested for extensive post-transcriptional editing. Only a small number of the NK cell transcripts identified were correlated with chronic HCV infection/replication indicating that inherent transcriptional activity prevails over environment effects such as viral infection.

**Conclusions:**

Collectively, inherent/genetic modulation of NK cell transcription is involved in setting the path to divergent treatment outcomes and could become useful to therapeutic advantage.

**Electronic supplementary material:**

The online version of this article (doi:10.1186/s12967-015-0428-x) contains supplementary material, which is available to authorized users.

## Introduction

Natural Killer (NK) cells were originally regarded as a component of first-line innate immune defences against invading pathogens and tumors [1]. This view has however considerably evolved to indicate that NK cells have higher functional complexity in the regulation of other innate and adaptive immune functions [2]. NK cell function relies on balanced signaling through inhibitory and activating receptors expressed on their surface [3,4] but a high degree functional variability exists due to inter-individual differences in inhibitory and activating receptor expression, resulting in 30.000 up to >100.000 NK cell phenotypes [5].

Triggering of activating NK cell receptor(s) and co-receptors (Natural Cytotoxicity Receptors - NCRs, NKp46, NKp44, NKp30; NKG2D; Fcγ-R; DNAM-1; NKC2C) in the absence of overriding inhibitory signalling results in the induction of NK cell function, including cytotoxicity and cytokines production [6-10]. Differences in activating NK cell receptor (i.e. NKp46, NKp30, DNAM-1, NKG2C) surface density directly associates to changes in NK cell function including cytotoxic activity [7], crosstalk with other cells of the immune system [11-13], cytokines production in recall-like responses [14-16], and control of pathogens replication through direct interaction with infected cells [17-22]. In line with these observations, in vivo differences in activating receptor density on peripheral NK cells are associated with diverging clinical courses upon acute viral infection with hepatitis C virus (HCV) [19,21] and during chronic HIV-1 infection [23-25]. During chronic HCV infection, derangements in NK cell cytokines production are detected involving interleukin 10 (IL-10) and IFN-g production [26,27]. Interestingly, not only Killer-Ig-like Inhibitory Receptors (KIR) germline carriage [28] but also different expression of activating (i.e. NKp30,NKp46) and inhibitory (i.e. CD85j) NK cell receptors [29] are associated to diverging response.

There has been so far little scientific focus on the relationship between germline KIR carriage and actual KIR transcription/expression or on the relationship between activating receptor expression variability on the surface of NK cells, and underlying differences in their transcription. Thus, although converging evidences show that individual differences in activating NK cell receptor expression underlie diverging clinical courses, there has been so far no molecular characterization at the transcriptional level to support this view.

To address this issue, we studied phenotypic and transcriptional characteristics of purified NK cells in patients chronically infected with HCV genotype-1 virus who were subsequently treated with *PEG*-*IFN*α*/RBV* showing divergent clinical response to the treatment with either NR or SVR*.* In the present work we show that in chronically HCV-infected patients different baseline NK cell transcriptional characteristics accompany and correspond to different surface marker phenotypes and diverging clinical response to treatment.

## Material and methods

### Patients and blood samples

#### Training and validating set of HCV-1 patients

Nineteen patients chronically infected with HCV (HCV-1) (n = 9 as part of the training cohort and n = 10 as part of the validating cohort) followed up within program for surveillance and treatment at the Hepatology Unite, University of Genoa, Italy. Patients with HIV coinfection or advanced liver involvement, including cirrhosis and HCC were excluded. All patients gave full informed consent to treatment and to observational sampling. Patients were treated with pegylated IFN-a (180 g/ml) and Ribavinin (600-1200 mg/day according to weight) (*PEG*-*IFN*α*/RBV, PR)* and followed up for 48 weeks post treatment according to Italian treatment guidelines. HCV viral load was assessed at baseline and after 4 and 12 weeks of treatment to confirm early virus clearance. SVR was defined as persistent HCV RNA negative by Amplicor HCV Monitor (Roche, Milan, Italy) at end of treatment and beyond 6 months after stopping treatment. Non-responder patients (NR) included null-responders, partial responders, and relapsers according to viremia kinetics on treatment. HCV genotype was determined before treatment in all patients with the INNO-LiPA HCV II kit (Bayer Diagnostics, Emeryville, CA, USA). Only patients with genotype I were evaluated. The samples were divided in a training set and a validating set before analysis began. Peripheral blood (20 ml) was collected before *PEG*-*IFN*α*/RBV* treatment and used for PBMCs isolation by Ficoll density gradient centrifugation. PBMCs were further used for DNA and NK cells isolation as well as flow cytometer analysis.

#### Healthy donors and reverse validating group of patients

Peripheral blood (60 ml) derived from 7 healthy donors (HD) and 8 chronically infected HCV patients used in the reverse validation approach (CV-HCV) was collected at the Department of Transfusion Medicine, Clinical Center, National Institutes of Health with IRB approval. For CV-HCV patients’ genotype assessment, INNO-LiPA HCV II kit (Bayer Diagnostics, Emeryville, CA, USA) was used. Only patients with genotype I were evaluated.

Pheripheral blood was used for PBMCs isolation by Ficoll density gradient centrifugation.

PBMCs were further used for NK cells isolation as below described. Detailed information about all patients used in the study is reported in Table 1.

**Table 1:** **Patients information used in the whole study**

	**Training group**		**Validating group**		**Healthy donors**	**Reverse validating group**	**Total patients**
**HCV Treatment Response**	**5 NR**	**4 SVR**	**5 NR**	**5 SVR**	**NA**	**NA**	**n=19**
**Caucasian**	**n=5**	**n=4**	**n=5**	**n=5**	**n=7**	**n=8**	**n=34**
**HCV genotype 1**	**n=5**	**n=4**	**n=5**	**n=5**	**NA**	**n=8**	**n=28**
**HCV Treatment naïve**	**n=5**	**n=4**	**n=5**	**n=5**	**n=7**	**n=8**	**n=34**
**Age mean±SD**	**44,2±18,36**	**32,3±14,43**	**60±7,8**	**51±6,4**	**45±4**	**48±16**	**n=34**
**Viral Load mean ±SD**	**3256750 ± 2672345,209**	**646000 ± 234921,3**	**3076667 ± 2438080**	**1506000 ± 1887003**	**NA**	**1908796 ± 1457469**	**n=27**
**ALT mean±SD**	**90,25±41,83**	**48±7**	**66 ± 55**	**116±90,23**	**NA**	**72±65**	**n=27**
**F1**	**n=2**	**n=2**	**n=0**	**n=2**	**NA**	**n=2**	**n=8**
**F2**	**n=0**	**n=1**	**n=0**	**n=1**	**NA**	**n=2**	**n=4**
**F3**	**n=0**	**n=1**	**n=3**	**n=1**	**NA**	**n=3**	**n=9**
**F4**	**n=3**	**n=0**	**n=2**	**n=1**	**NA**	**n=3**	**n=9**
**Total patients**	**n=9**		**n=10**		**n=7**	**n=8**	**n=34**

Legend: NR = Non responders; SVR=sustained virologic responders; HD=Healthy donors. SD= Standard Deviation. Viral Load values are expressed as viral copies (CP)/ml; ALT values are expressed as UI/ml. Fibrosis in patients with HCV was staged according to the Metavir classification, which ranges from F0 to F4 (F0 = no fibrosis; F1 = portal fibrosis without septa; F2 = portal fibrosis with few septa; F3 = portal fibrosis with many septa; and F4 = cirrhosis).

### DNA isolation and IL28 rs12979860 polymorphism screening

DNA isolation from PBMCs derived from validating and training groups of HCV patients was performed by using Fujifilm’s Quickgene DNA Whole Blood kit (Fujifilm Medical Systems USA, Stamford, CT). DNA was used for screening of IL28B *rs12979860* polymorphism by using TaqMan® SNP Genotyping Assays (Life Technologies, Grand Island, NY) following manufacturer’s instructions. Genetic correlation of IL28B *rs12979860 CC* polymorphism with diverging clinical response was evaluated by Fisher’s two tails exact test by using Graph Pad Prism (San Diego, CA, USA).

### NK cells isolation from PBMCs

NK cells isolation from PBMCs was conducted via magnetic-bead associated cell sorting, using Miltenyi’s Human NK cell isolation (Miltenyi Biotec, San Diego, CA) kit for negative selection.

### Immunofluorescence analysis and Abs

Following isolation, the purity of NK cells was evaluated in all patients (SVR, NR, HD and CV-HCV) by immunofluorescence analysis resulting in CD3^−^CD14^−^CD19^−^CD56^+^ gate >97%.

In training group of SVR and NR patients analysis of NKp30 and NKG2D expression was evaluated by immunofluorescence on CD56 + CD3-CD14-CD19- PBMCs.

Because of increased number of isolated NK cells derived from the 60 ml of peripheral blood, in CV-HCV patients the expression of NKp30 and NKG2D expression was evaluated by immunofluorescence directly on purified NK cells. The correlation of NKp30 and NKG2D expression with clinical response was performed by Mann–Whitney *u* tests for unpaired datasets by using MedCalc Software (Mariakerke, Belgium) and Stat graphics centurion (Warrenton, VA, USA).

The following panel of mouse anti-human mAbs was used for all immunofluorescence screenings: anti-CD3, −CD19, −CD14 allophycocyanin (APC)-conjugated (BD PharMingen, San Jose, CA, USA), anti-CD56 PeC7-conjugated (Immunotech-Coulter Marseille, France). Anti-NKp30 and NKG2D were in house produced and gently provided by A. Moretta. Briefly, cells were incubated with primary mAbs followed by PE- or FITC-conjugated anti-isotype-specific goat anti-mouse secondary reagents (BD PharMingen, San Jose, CA, USA). Direct staining was performed by fluorochrome-conjugated mAbs as a third step. For cytofluorimetric analysis, cells were gated using forward and side light scatter parameters (FACSCantoII, BD, Mountain View, CA, USA) and 10,000 events were always acquired. Data were analysed using FlowJo (Tree Star, Inc.).

### Gene expression array

Total RNA was purified from NK cells derived from HD, SVR, NR and CV-HCV individuals by using miRNeasy minikit (Qiagen, Germantown, MD,USA) according to the manufacture’s protocol. RNA quality and quantity was estimated using Nanodrop (Thermo Scientific, Waltham, MA, USA) and Agilent 2100 Bioanalyzer (Agilent Technologies, Palo Alto, CA, USA). First- and second-strands cDNA were synthesized from 30 ng of total RNA by using Nugen Ovation Pico WTA System V2 (Nugen technologies, San Carlos, CA, USA) and following manufacturer’s instructions. cDNAs were fragmented and biotinylated by using Nugen Encore Biotin Module (Nugen technologies, San Carlos, CA, USA) and hybridized to the GeneChip Human Gene 1.0 ST Arrays (Affymetrix, Santa Clara, CA, USA). The arrays were washed and stained on a GeneChip Fluidics Station 450 and scanned by GeneChip Scanner 3000 (Affymetrix, Santa Clara,CA, USA). The global gene expression profiling of NK cells was analyzed using BRBArrayTools developed by the Biometric Research Branch, NCI (http://linus.nci.nih.gov/BRB-ArrayTools.html) and Partek Genomics Suite (St Louis, MO). Functional analysis was performed using the Ingenuity Pathway Analysis system (IPA), a web-based software application for the analysis, integration, and interpretation of data derived from ‘omics’ experiments, such as RNAseq and microarrays. Powerful algorithms identified regulators, relationships, mechanisms, functions, and pathways relevant to changes observed in an analyzed dataset.

## Results

### Molecular organization of NK cells during HCV infection is associated with treatment outcome

Purified NK cells from prospectively collected cryopreserved PBMCs on a set of 9 sequential HCV patients starting treatment with *PEG*-*IFN*α*/RBV* for 48 weeks were evaluated first.

Gene expression arrays were employed for global transcriptional profiling of the purified NK cells and stratified for comparison according to treatment outcome (5 non-responder (NR) and 4 sustained virological responder (SVR)). Purified peripheral NK cells from 7 healthy donors (HD) were used as control.

**Figure 1:**
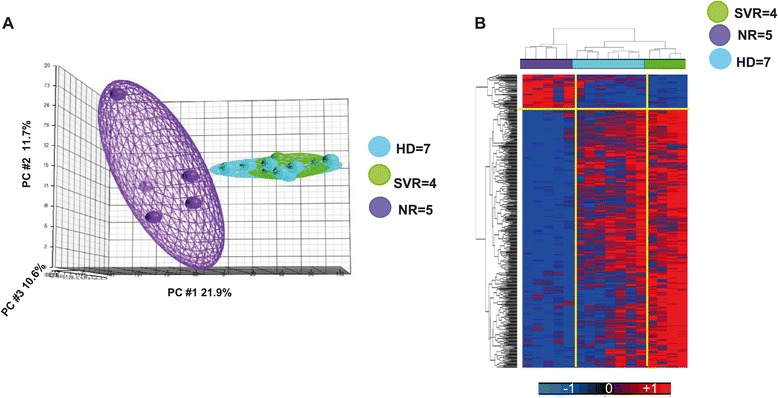
**Trascriptional profile of NK cells derived from HCV infected patients with diverging treatment response. A)** PCA analysis conducted on whole gene dataset expressed by NK cells of Healthy Individuals (HD), baseline responders (SVR) and non-responders (NR) HCV-1 patients. **B)** Supervised cluster based on 476 genes derived from Student’s t Test (cut off p_2_ value ≤ 0.005, FC >1.5) SVR vs. NR.

Principal Component Analysis (PCA), a method that identifies gene-expression patterns (principal components) that best explain variance across a data set, was performed on the whole dataset (33,304 transcripts) revealing that pre-treatment transcriptional patterns of purified NK cells were clearly heterogeneous. In particular, transcriptional profiles from patients with subsequent SVR on treatment clearly segregated apart from those of NR patients. Surprisingly, despite the presence of chronic HCV replication, a similar NK transcriptional pattern was found for SVR and HD (Figure 1A).

Prior to treatment our analysis identified 476 transcripts differentially expressed by NK cells in patients with SVR compared to NR (cut off p_2_ value ≤ 0.005; Fold Change >1.5). Among differentially expressed transcripts, 416 were up-regulated (Additional file 1: Table S1) and 60 were down-regulated (Additional file 2: Table S2) in patients who subsequently responded to treatment.

A supervised clustering was performed using these 476 transcripts in SVR and NR patients. In order to evaluate the trend of expression of the 476 differentiating genes also in healthy donors, the supervised clustering was conducted also by including the value of expression of the 476 genes in healthy individuals. The results demonstrated that the expression of genes differentially expressed in SVR vs NR patients are instead similarly expressed in SVR vs. HD.

**Figure 2:**
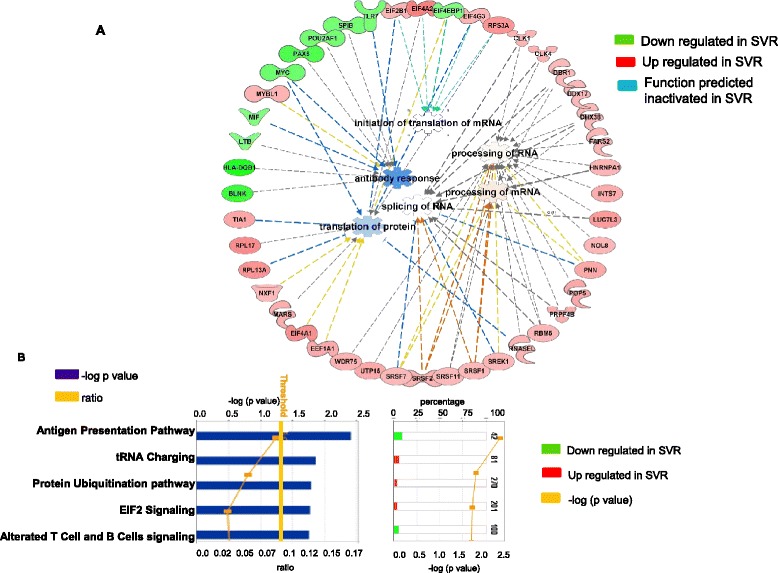
**Functional analysis conducted on genes differentially expressed in HCV patients with diverging treatment response. A)** IPA analysis conducted on the 476 transcripts differentially expressed in SVR vs. NR patients showed in the top network that molecules involved in mRNA processing and protein synthesis (such as SRSF molecules) are up regulated (in red) in SVR samples. **B)** Top 5 significant ranking significant (threshold p value < 0.05) canonical pathways conducted on the 476 transcripts reveled an up regulation of ubiquitination pathways as well as tRNA charging pathways in SVR patients. On the contrary, molecules involved in antigen presentation pathways are showed to be down regulated in SVR patients. The *p* value for each pathway is indicated by the blue bar and is expressed as –1 times the log of the *p* value. The yellow line represents the ratio of the number of genes in a given pathway that meet the cutoff criteria divided by the total number of genes that make up that pathway.

Functional analysis performed by Ingenuity Pathways Analysis (IPA) of the 476 NK genes showed that the transcripts which correlated most significantly with treatment outcome were those coding for molecules involved in post-transcriptional modification of RNA/protein trafficking and those associated with HLA class II signaling (Figure 2A). In particular, purified NK cells from SVR patients displayed an increased expression of genes involving post transcriptional regulation and alternative splicing such as small nucleolar RNAs (snoRNA, known also as SNORD). Indeed, the top down-regulated transcripts in NR patients were snoRNAs. Although the exact mechanism of action of snoRNA is still undefined, snoRNAs have been shown to orchestrate the splicing of RNA [30,31] and the folding of pre-rRNA necessary for correct processing and ribosomal protein assembly in the nucleolus [32]. In addition to snoRNAs, other molecules involved in RNA processing such as serine/arginine-rich splicing factors (SRSF) also were found overexpressed in SVR. The findings were confirmed by functional analysis conducted on the 476 genes by using IPA. In particular the results revealed an up regulation of molecules involved in mRNA processing and protein translation functions in SVR patients (Figure 2A). Similar results were obtained by looking at first significant (p value < 0.05) canonical pathways in which molecules involved in ubiquitination pathways and RNA charging pathways are shown up regulated in SVR patients (Figure 2B). These observations further support the presence of relevant differences in the arrangement of RNA processing and protein synthesis machinery of NK cells in different patient groups and different treatment outcomes. No overall difference was observed in the interferon-stimulated transcript pathway.

### Protein expression of NKp30 and NKG2D correlates with divergent treatment responses during HCV infection

**Figure 3:**
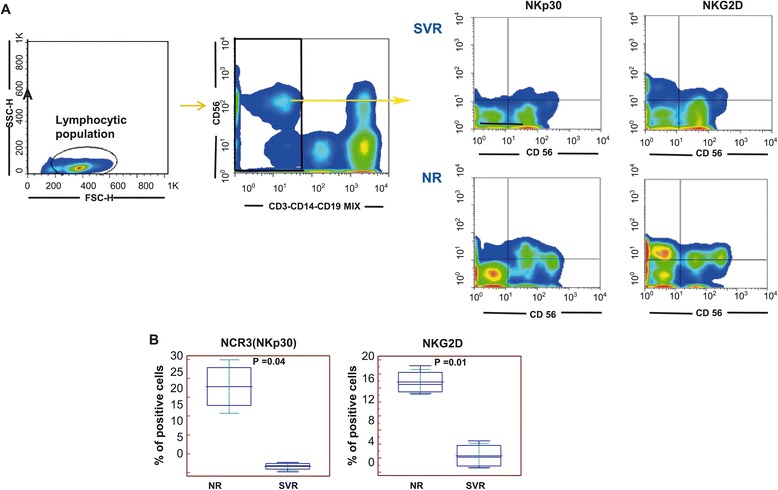
**Correlation of activating NK cell receptor (NKp30 and NKG2D) expression with divergent treatment responses during HCV infection. A)** Representation of gating strategy adopted for the evaluation of NKp30 and NKG2D expression in the CD56 + CD3-CD19-CD14- PBMCs derived from training SVR and NR patients. **B)** Further correlation (Mann Whitney u test) with INFα + rivabirin treatment response showed a significant up regulation of NKp30 and NKG2D in NR. Results obtained for NKp30 confirmed previous observations [29].

We next examined possible differences in surface molecule expression/phenotype by flow cytometry to further evaluate their transcriptional correlates (Figure 3A). Flow cytrometric analysis of PBMCs in the training group of patients showed that SVR patients had decreased proportions of CD56 + NKp30+ (Mann–Whitney *U*-test p = 0.04) and of CD56 + NKG2D+ (Mann–Whitney *U*-test p = 0.01) (Figure 3B). No differences were observed for other NCRs (data not shown). These results were in line with previous work showing that NKp30 is differentially expressed on CD56+ cells from patients who clear HCV upon INF-a + ribavirin treatment vs. non-responder patients [29].

Despite their low surface expression, the molecular expression of NKp30 was not significantly different in SVR vs. NR patients (p = 0.88, Student’s t test). Indeed, the NKG2D transcript was found to be up-regulated in patients with SVR (p value 0.003). The decreased protein surface expression of NKG2D with increased transcript presence at baseline in SVR patients suggested a “protective” role played by posttranscriptional events. This is in line with other reports and confirms that the surface molecule expression of NKG2D is regulated by extensive posttranscriptional mechanism(s) [33].

### Validation of the SVR: NR gene set transcriptional profile in independent cohorts of HCV patients

The significant individual differences observed in the transcriptional activity of purified NK cells in HCV-patients after patient stratification according to response to treatment raised the question of whether this was the result of a selection bias in patients entering in a single treatment trial.

**Figure 4:**
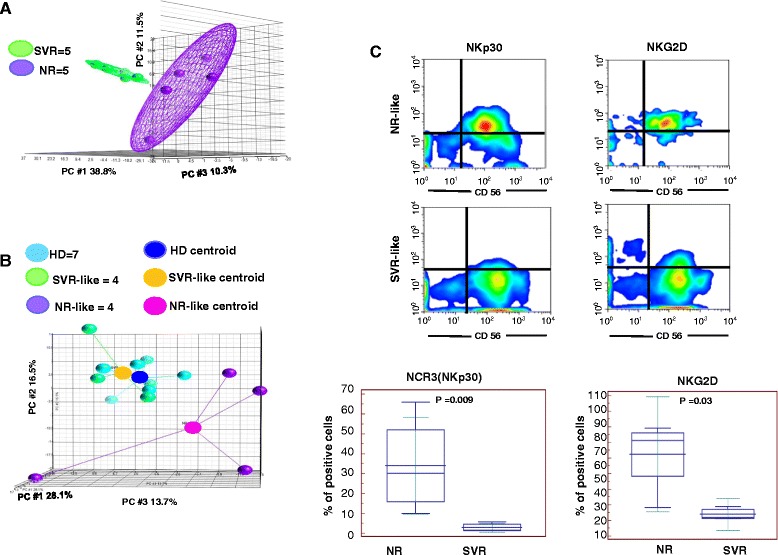
**The SVR: NR gene set transcriptional profile is validated in independent cohorts of HCV patients. A)** PCA analysis performed on the independent group of patients by using the 476 genes associated with treatment response in the training set of patients; **B)** PCA analysis performed on 8 treatment naïve chronically infected HCV patients (CH-HCV) by using the 476-transcript signature segregated the CH-HCV patients according a SVR-like and NR-like. Because of the high heterogeneity of NK profile derived from NR-like patients, the PCA was conducted by adding a centroid for each group of patients. **C)** The expression of NKp30 and NKG2D was evaluated on purified NK cells showing to be higher in NR-like patients as previously reported to occur for NR patients [29,34].

We therefore studied whether the 476 gene-signature set identified in NK cells from NR vs. SVR patients would also segregate in chronically infected patients followed independently in another cohort of patients. To this end, independent gene expression analysis was performed on purified NK cells from PBMCs derived from 5 NR and 5 SVR patients from University of Genoa, Italy. Samples were obtained prior to treatment with PEG-*IFN/RBV.* PCA analysis was performed on this validating group of patients by using the 476 transcripts identified in the training set of patients, and confirmed that this gene set could efficiently differentiate SVR from NR patients in an independent cohort (Figure 4A).

Next, we studied a new group of 8 treatment-naive chronically infected HCV-patients (CV-HCV) followed up in a natural history study in North America (Clinical Center, NIH, MD, USA). Gene expression profiling was performed on their purified NK cells derived from PBMCs. PCA analysis performed on the 476-transcript signature showed segregation of these patients into two groups. In particular, PCA analysis showed that one group of patients (n = 4) had transcript signatures very similar to that obtained in healthy donors and in patients that had sustained virologic responses in the treatment cohort shown in Figure 1A. The remaining patient group (n = 4) was clearly different from HD and those with SVR and was highly heterogeneous. We hypothesized that this group would behave as NR or as relapsers when treated with a standard PR treatment regimen (Figure 4B). To verify this assumption we reverse validated data (thus *reverse validating approach*) by studying the expression of NKG2D and NKp30 on their peripheral NK cells after gene signature stratification. In the reverse validating approach, cytofluorimetric analysis of CD3^−^CD56^+^NKp30^+^ and CD3^−^CD56^+^NKG2D^+^ showed a significantly lower expression of NKp30 and NKG2D in patients with an NK cell transcriptional profile very comparable to HD (SVR-like group) and conversely an increased expression in NR-like group (Figure 4C). This finding thus indicates that the identified transcriptional NK cell pattern in treated patients is also reflected in a different group of treatment-naive patients and differentiates those with enhanced potential for SVR [35] from those who would not respond to peg-IFNα + ribavirin treatment. Taken together, these observations show that in any given setting at least two gene signatures may be identified in HCV patients independent of the clinical setting and location.

### Inherent HCV-independent transcriptional regulation of NK cells underlies divergent clinical responses to treatment with IFN-a and ribavirin

**Figure 5:**
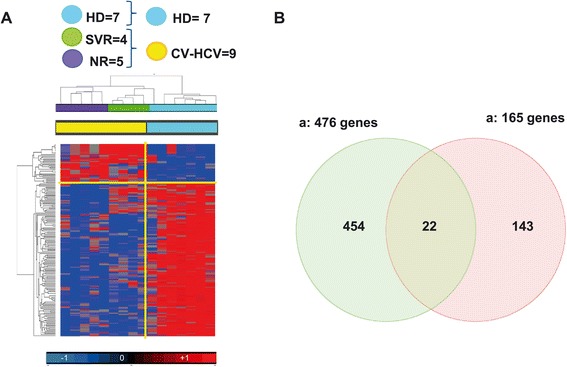
**Analysis of pre-treatment NK cells trascriptome according to the level of HCV viremia. A)** Supervised cluster based on 165 genes derived from Student’s t Test Chronic Viraemic HCV patients (CV-HCV) vs. HD (Cut off p value <0.005; FC > 1.5). **B)** Venn diagram between the 476 genes able to differentiate NR vs. SVR patients and the 165 genes associated with viremia. Among the genes able to segregate the SVR from the NR, only 22 genes (5%) were affected by viremia.

The level of HCV replication prior to treatment is likely to affect baseline NK cell transcriptional activity and function. To determine the extent of this interaction, we next studied whether the differential expression of the 476 NK transcripts could be affected by HCV viremia. To this end, we compared the transcriptional profiles of purified peripheral NK cells from healthy donors (HD) and from chronically viremic HCV-infected patients (CH-HCV). Comparative analysis identified 165 transcripts differentially expressed by NK cells in HD vs. CH-HCV (Student’s T test p_2_ v ≤ 0.005, FC ≥1.5) (Figure 5A). Among the most up-regulated transcripts in CH-HCV patients, were molecules involved in interferon signaling (Additional file 3: Table S3), whose expression has been previously reported to be increased in the course of HCV infection in these patients [36]. A Venn diagram showed that among the 165 transcripts, only 22 transcripts (5%) were included in the set of 476 transcripts previously found to differentiate SVR from NR patients (Figure 5B). Conversely, 95% of the genes whose transcription is affected by HCV were not included in the list of those that are differentially expressed among patients according to their response to treatment.

These results thus show that only a negligible fraction of the NK cells transcripts that are modulated by chronic HCV replication are able to clinically segregate NR from SVR patients. Rather, inherent HCV-independent transcriptional regulation of NK cells underlies divergent clinical responses to treatment with IFN-a and ribavirin.

### IL28B *rs12979860* polimorphism doesn’t clearly distinguish patients with widely diverging outcome in our series of patients

Genetic polymorphisms near the *IL28B* gene, encoding interferon-λ-3 (IFN-λ-3), gene have been previously associated with response to treatment [37].

In order to investigate this aspect in our dataset, IL28B *rs12979860* polymorphism screening was performed by RT-PCR using DNA extracted from PBMCs of both the training and validating groups of HCV-1 patients. These results showed that IL28B IL28B *rs12979860* polymorphism was not significantly associated with SVR in our series of patients (F test p =0.99) (Additional file 4: Table S4).

## Discussion

In the present work we studied peripheral NK cell transcriptional diversity in patients chronically infected with HCV by microarray analysis and flow cytometry showing that inter-individual NK cells baseline differences exist and may predict subsequent diverging responses to treatment in interferon-based regimens. Relevant inter-individual differences in mature peripheral NK cell phenotype have been described previously in patients with chronic infections and divergent clinical courses or treatment responses [24,28,29]. In these instances, NK cell receptors are poorly expressed but are inducible in patients controlling HIV infection spontaneously (NKp30, NKp46) and in those clearing HCV upon peg-IFNa/ribavirin treatment (NKp30). Although differences in individual surface expression are the consequence of individual nuances in transcriptional regulation of cell transcription, thus far these have received little attention. The present characterization represents, to our knowledge, the first attempt to shed light on this aspect during chronic HCV infection. In this study, PCA analysis identified two divergent NK signatures that stratified patients according to subsequent treatment response to IFN (SVR vs. NR). This observation is in line with transcriptional differences in NK-cell specific genes observed in patients with recurrent vs. nonrecurring breast cancer [38,39] and in GIST (Gastrointestinal Chronic Tumor) [40], where NK cell signatures of activating receptor transcription in tumor infiltrating lymphocytes are associated with nonrecurring disease. Previous work showed that in patients who will respond to IFN treatment, NKp30 expression on peripheral NK cells is significantly lower compared to non-responding patients. Here we confirmed this observation and extended it also to NKG2D expression. Surprisingly, the differences in transcriptional activity of peripheral NK cells among SVR and NR patients did not directly support some of the phenotypic differences observed (such as the decreased surface expression of NKp30 and NKG2D). These findings suggest that differences in the expression of NK cells derived from patients with different treatment outcomes might be affected by extensive posttranscriptional regulation. Indeed, there was a significant upregulation of SNORDs and regulatory small RNAs in SVR patients, raising the possibility of relevant posttranscriptional regulation in SVR, but not in NR where small noncoding RNAs (ncRNAs) expression is lower. These data indicate that the relevance of the non-coding genome is not limited to microRNA (miRNA) expression and function, and that other ncRNAs, (i.e.:snoRNAs) are involved in the shaping of inherently different NK immune responses resulting in a different arrangement of the protein synthesis machinery. Whether this observation may be exploited for clinical predictive evaluation of response to treatment, or for other clinical purposes in view of the advent of new HCV-specific regimens, is still undefined. Large quantification and clinical validation studies would be needed to confirm this possibility. Along this line, the finding that HD tightly clustered with SVR based on the expression of genes differentially expressed between SVR vs NR may be surprising. Indeed considering the 40-60% chance of chronically infected patients of responding to PR treatment, and given the 30% probability of any acutely infected patient to spontaneously clear the infection, it can be expected that HD are particularly enriched in subjects either spontaneously clearing the infection (30%) or responding to treatment (50% of 70% i.e. 35%). This could mean that up to 70% of any HD cohort could share the SVR signature. Therefore, the observed HD-SVR clustering may be explained by the small sample size, and needs to be addressed further in larger HD cohorts. In addition to the down-regulation of molecules involved in post transcriptional events we observed a very significant up-regulation of HLA class II transcripts in NK cells from NR patients. This is in line with the crucial role played by HLA class II molecules in treatment-induced-clearance of HCV and is supported by previous reports showing that SNPs marking HLA-class II (i.e. Homozygous C1 supertype) are strongly associated with the outcome of Hepatitis C Virus Infection [41,42]. Moreover, the present results additionally suggest that not only qualitative (i.e. homozygous C1 supertype) but also quantitative (i.e. increased HLA class II transcription) differences in NK cell HLA are relevant and may efficiently discriminate patients with diverging treatment responses and disease courses.

Surprisingly, none of the genes found to differentiate patients with diverging clinical response to therapy were related to the IL28B polymorphism, nor an IL28B CC homozygosis was significantly associated with SVR in our series of patients. These results should however be considered in the perspective of an analysis deriving from a small cohort of patients as opposed to large population statistics needed for correct SNPs evaluation.

Comparison of microarray transcriptional regulation in all HCV patients to a group of healthy donors allowed investigation of the extent to which differences in NK cell transcriptional regulation reflect the effects of chronic HCV infection per se. Indeed, HCV replication affects peripheral NK cell transcriptional activity, as shown by the presence of 165 differentially regulated genes. Notably, only 22 of these 165 transcripts were included in the set of 476 genes that allowed differentiation between treatment responders and non-responders. Therefore, the transcriptional (and phenotypic) differences observed here in SVR vs. NR patients reflect a predominantly (95%) inherent regulation of treatment response in HCV-infected patients, with only a minor influence of HCV replication.

In the present analysis we identified at least two inherent/genetic NK cell signatures when analyzed by PCA analysis and stratified by clinical response to treatment. This analysis, however, does not exclude that stratification by other parameters might provide additional insights into NK activity. In contrast to mass cytometry, one of the limits of the present microarray analysis and standard flow cytometry is the need to consider mean values in a group disregarding the many nuances of NK cell molecule transcription/expression. For example, additional transcriptional groupings may be observed when analyses are performed on different NK cell subsets (i.e. CD56bright, CD56dim, CD56-) or on NK cells expressing NKG2C, or residing in the liver during chronic HCV infection.

## Conclusions

This work provided a transcriptional context for known and previously unknown molecular aspects of NK cell identity and function. By delineating the genome-wide repertoire of gene expression of NK cells in HCV patients and using diverging response to treatment regimens as dissecting tool, we confirm that different gene signatures underlie previously described phenotypic differences. A multiplicity of transcriptional regulators is involved in this diversity of innate NK cell responses with inherent genetic determinants predominating over environmental (i.e. HCV) factors.

## Additional files

Additional file 1: Table S1. Student’s T test SVR vs. NR (p < 0.005; FC >1.5). List of 4 16 transcripts up regulated in SVR patients. Transcripts are ordered based on descending Fold Change (FC). Additional file 2: Table S2. Student’s T test SVR vs. NR (p < 0.005; FC < −1.5). List of 60 transcripts down regulated in SVR patients. Transcripts are ordered based on descending Fold Change (FC). Additional file 3: Table S3. Student’s T test CV-HCV vs. HD (p < 0.005; FC > 1.5 or Fc < −1.5). List of 165 genes up-regulated and down regulated in CV-HCV compared to HD. Transcripts are ordered based on descending Fold Change (FC). Additional file 4: Table S4. Screening of IL28B *rs12979860* polymorphism in the training and validating groups of HCV patients.
