# Supplementary material for: Inherent transcriptional signatures of NK cells are associated with response to IFNα + rivabirin therapy in patients with Hepatitis C Virus
Source: J Transl Med. 2015 Mar 1;13:77. doi: 10.1186/s12967-015-0428-x (PMC4353456; doi:10.1186/s12967-015-0428-x)
Supplement: Additional file 1: Table S1. — Student’s T test SVR vs. NR (p < 0.005; FC >1.5). List of 4 16 transcripts up regulated in SVR patients. Transcripts are ordered based on descending Fold Change (FC). [file 12967_2015_428_MOESM1_ESM.docx]

| **Gene** | **P value** | **FC** | **Gene** | **P Value** | | **FC** | **Gene** | **P value** | **FC** | **Gene** | **P value** | | **FC** |
| --- | --- | --- | --- | --- | --- | --- | --- | --- | --- | --- | --- | --- | --- |
| **ANKRD20A11P** | **0.0008** | **15.29** | **TIA1** | | **0.0000** | **1.90** | **ROCK2** | **0.0016** | **1.66** | **RBM5** | | **0.0005** | **1.56** |
| **SNHG1** | **0.0000** | **4.54** | **MYSM1** | | **0.0000** | **1.90** | **EPM2AIP1** | **0.0000** | **1.66** | **ALG6** | | **0.0009** | **1.56** |
| **SNORD28** | **0.0004** | **4.39** | **BTAF1** | | **0.0000** | **1.89** | **ARAP2** | **0.0001** | **1.66** | **MACF1** | | **0.0001** | **1.55** |
| **SNORD38B** | **0.0000** | **4.34** | **LTV1** | | **0.0000** | **1.89** | **TGDS** | **0.0006** | **1.66** | **FBXO38** | | **0.0025** | **1.55** |
| **SNORD59B** | **0.0019** | **4.13** | **ZNF300** | | **0.0022** | **1.88** | **ACRC** | **0.0016** | **1.66** | **C10orf118** | | **0.0010** | **1.55** |
| **SNORD59A** | **0.0001** | **3.61** | **TUBE1** | | **0.0006** | **1.88** | **MTX2** | **0.0024** | **1.66** | **NPEPPS** | | **0.0001** | **1.55** |
| **SNORA33** | **0.0000** | **3.23** | **IL11RA** | | **0.0000** | **1.87** | **ZNF75A** | **0.0011** | **1.65** | **PPFIA1** | | **0.0011** | **1.55** |
| **AMY2A** | **0.0000** | **3.22** | **LINC00299** | | **0.0001** | **1.87** | **GOLGA8M** | **0.0019** | **1.65** | **TRMT10B** | | **0.0005** | **1.55** |
| **RNU6-79P** | **0.0018** | **3.17** | **FASTKD3** | | **0.0005** | **1.87** | **DNAH1** | **0.0006** | **1.65** | **NOL8** | | **0.0001** | **1.55** |
| **LOC101059935** | **0.0006** | **3.13** | **RABGGTB** | | **0.0001** | **1.86** | **CEP78** | **0.0003** | **1.65** | **LARS2** | | **0.0006** | **1.55** |
| **ANKRD20A8P** | **0.0002** | **3.07** | **GPR89B** | | **0.0000** | **1.86** | **ZNF502** | **0.0030** | **1.65** | **ZFP3** | | **0.0041** | **1.55** |
| **SNORD20** | **0.0016** | **3.06** | **NSUN5P1** | | **0.0000** | **1.86** | **IRAK4** | **0.0003** | **1.65** | **CNTRL** | | **0.0006** | **1.55** |
| **SNORD30** | **0.0000** | **3.04** | **SNORA7B** | | **0.0010** | **1.86** | **RPE** | **0.0018** | **1.65** | **UBR5** | | **0.0000** | **1.55** |
| **AHSA2** | **0.0000** | **2.99** | **OSGEP** | | **0.0000** | **1.85** | **ATP6V1C1** | **0.0030** | **1.65** | **ZNF321P** | | **0.0008** | **1.55** |
| **SNORD54** | **0.0029** | **2.95** | **PNN** | | **0.0005** | **1.85** | **AGPAT4-IT1** | **0.0033** | **1.65** | **TAF11** | | **0.0000** | **1.55** |
| **AMY2B** | **0.0029** | **2.92** | **POP5** | | **0.0019** | **1.85** | **MCCC1** | **0.0006** | **1.65** | **GTF3C3** | | **0.0012** | **1.55** |
| **TAF1D** | **0.0016** | **2.86** | **PTCD3** | | **0.0001** | **1.85** | **SRSF2** | **0.0003** | **1.64** | **IWS1** | | **0.0032** | **1.55** |
| **GSAP** | **0.0001** | **2.74** | **PAN2** | | **0.0003** | **1.85** | **METTL17** | **0.0004** | **1.64** | **PHKG2** | | **0.0000** | **1.54** |
| **SNORD82** | **0.0000** | **2.74** | **ANKRD36C** | | **0.0002** | **1.85** | **WDR60** | **0.0005** | **1.64** | **PARP6** | | **0.0001** | **1.54** |
| **EIF4A2** | **0.0001** | **2.72** | **SLTM** | | **0.0002** | **1.83** | **PRPF39** | **0.0037** | **1.64** | **SPATA20** | | **0.0041** | **1.54** |
| **SNORD50B** | **0.0001** | **2.72** | **MYO15B** | | **0.0010** | **1.83** | **ZNRD1-AS1** | **0.0007** | **1.64** | **CARF** | | **0.0041** | **1.54** |
| **ORM2** | **0.0012** | **2.72** | **PUS10** | | **0.0007** | **1.83** | **RBL2** | **0.0015** | **1.64** | **DDX17** | | **0.0002** | **1.54** |
| **SNORD57** | **0.0000** | **2.67** | **CREBZF** | | **0.0001** | **1.82** | **POLI** | **0.0000** | **1.64** | **SREK1** | | **0.0018** | **1.54** |
| **NMRK1** | **0.0001** | **2.65** | **BTN3A1** | | **0.0012** | **1.82** | **GABPA** | **0.0025** | **1.64** | **RBM28** | | **0.0007** | **1.54** |
| **DDX18P1** | **0.0001** | **2.65** | **CDC16** | | **0.0000** | **1.82** | **FCHO2** | **0.0023** | **1.64** | **FAM219B** | | **0.0012** | **1.54** |
| **CCDC146** | **0.0006** | **2.64** | **NUP107** | | **0.0022** | **1.81** | **COG4** | **0.0011** | **1.64** | **FAM188A** | | **0.0006** | **1.54** |
| **ANKRD36** | **0.0000** | **2.61** | **EEF1A1** | | **0.0005** | **1.81** | **TCTEX1D2** | **0.0047** | **1.64** | **LOC646358** | | **0.0041** | **1.54** |
| **OTTHUMG00000002215** | **0.0001** | **2.60** | **UTP15** | | **0.0034** | **1.81** | **WDR75** | **0.0005** | **1.64** | **SMAD5** | | **0.0049** | **1.54** |
| **HSPA8** | **0.0014** | **2.57** | **TTC32** | | **0.0012** | **1.80** | **HSD17B7** | **0.0005** | **1.64** | **NEK1** | | **0.0013** | **1.54** |
| **ACTG1P4** | **0.0000** | **2.54** | **SNORD116@** | | **0.0006** | **1.80** | **PIK3R4** | **0.0015** | **1.64** | **NSMAF** | | **0.0034** | **1.53** |
| **RABGGTB** | **0.0009** | **2.53** | **TMEM14E** | | **0.0040** | **1.80** | **KLHDC2** | **0.0008** | **1.64** | **PEX11B** | | **0.0045** | **1.53** |
| **HSD17B7P2** | **0.0001** | **2.50** | **POLG2** | | **0.0008** | **1.79** | **GPRASP1** | **0.0000** | **1.64** | **LOC100132099** | | **0.0034** | **1.53** |
| **TBRG4** | **0.0038** | **2.50** | **IFT80** | | **0.0020** | **1.79** | **HCFC2** | **0.0000** | **1.64** | **POLR2B** | | **0.0016** | **1.53** |
| **KLRC4** | **0.0033** | **2.47** | **NSUN6** | | **0.0045** | **1.79** | **ZNF33A** | **0.0009** | **1.64** | **LRRC37BP1** | | **0.0000** | **1.53** |
| **RPL17** | **0.0005** | **2.44** | **UBA7** | | **0.0005** | **1.79** | **HNRNPA1** | **0.0003** | **1.63** | **TRIP12** | | **0.0020** | **1.53** |
| **LOC101060026** | **0.0010** | **2.44** | **NGDN** | | **0.0001** | **1.79** | **CLK4** | **0.0027** | **1.63** | **MORC2** | | **0.0006** | **1.53** |
| **EIF4A1** | **0.0013** | **2.43** | **INTS7** | | **0.0001** | **1.78** | **CLK1** | **0.0004** | **1.63** | **MAP3K7** | | **0.0004** | **1.53** |
| **MOSPD2** | **0.0003** | **2.42** | **SNORA45** | | **0.0000** | **1.78** | **UBXN4** | **0.0002** | **1.63** | **EIF2S2P4** | | **0.0010** | **1.53** |
| **SNORD50A** | **0.0001** | **2.38** | **DLG5** | | **0.0001** | **1.78** | **MAP4K3** | **0.0017** | **1.62** | **PHIP** | | **0.0001** | **1.53** |
| **SNORD37** | **0.0008** | **2.36** | **NKTR** | | **0.0015** | **1.77** | **MYBL1** | **0.0013** | **1.62** | **RBM26** | | **0.0003** | **1.53** |
| **SNORA14B** | **0.0018** | **2.33** | **ASUN** | | **0.0000** | **1.77** | **PCYT1A** | **0.0003** | **1.62** | **TTC3** | | **0.0020** | **1.53** |
| **RPL13A** | **0.0012** | **2.32** | **AGL** | | **0.0049** | **1.76** | **THTPA** | **0.0002** | **1.62** | **CSPP1** | | **0.0015** | **1.53** |
| **RNF185** | **0.0031** | **2.30** | **HCRP1** | | **0.0040** | **1.76** | **RNASEL** | **0.0019** | **1.62** | **SRP54** | | **0.0001** | **1.53** |
| **CRIPAK** | **0.0015** | **2.29** | **ABCA5** | | **0.0002** | **1.76** | **DCAF17** | **0.0046** | **1.62** | **UBA3** | | **0.0007** | **1.53** |
| **RPS3A** | **0.0005** | **2.29** | **SLC4A1AP** | | **0.0001** | **1.75** | **JHDM1D** | **0.0003** | **1.62** | **RRAGB** | | **0.0038** | **1.53** |
| **SNORD116-8** | **0.0022** | **2.28** | **HNRNPU-AS1** | | **0.0006** | **1.75** | **FAM111A** | **0.0018** | **1.62** | **PCSK7** | | **0.0003** | **1.52** |
| **FAM27A** | **0.0038** | **2.26** | **ZNF131** | | **0.0015** | **1.75** | **TIMM21** | **0.0044** | **1.62** | **GNPAT** | | **0.0005** | **1.52** |
| **ADHFE1** | **0.0000** | **2.26** | **ACAD8** | | **0.0016** | **1.75** | **CDC7** | **0.0006** | **1.62** | **SACS** | | **0.0015** | **1.52** |
| **ZNF600** | **0.0001** | **2.24** | **ARPC4-TTLL3** | | **0.0000** | **1.75** | **PIGN** | **0.0011** | **1.61** | **RARS2** | | **0.0001** | **1.52** |
| **TRMT13** | **0.0000** | **2.23** | **INTS4L2** | | **0.0006** | **1.74** | **ZBED5** | **0.0001** | **1.61** | **DENND1B** | | **0.0046** | **1.52** |
| **VPS29** | **0.0032** | **2.22** | **NBEAL1** | | **0.0002** | **1.74** | **RASA2** | **0.0000** | **1.61** | **CHD1L** | | **0.0035** | **1.52** |
| **METTL3** | **0.0000** | **2.21** | **PRPF4B** | | **0.0006** | **1.74** | **HLTF** | **0.0020** | **1.61** | **ACADVL** | | **0.0045** | **1.52** |
| **NAPB** | **0.0000** | **2.21** | **TRMT11** | | **0.0026** | **1.74** | **DPYD** | **0.0005** | **1.61** | **CASP8AP2** | | **0.0004** | **1.52** |
| **ANKRD36B** | **0.0001** | **2.20** | **RRN3P1** | | **0.0002** | **1.74** | **UTP6** | **0.0006** | **1.61** | **KDM4C** | | **0.0003** | **1.52** |
| **PCGF3** | **0.0006** | **2.18** | **IQCB1** | | **0.0038** | **1.74** | **C10orf137** | **0.0000** | **1.61** | **SRSF11** | | **0.0007** | **1.52** |
| **LUC7L3** | **0.0001** | **2.17** | **MARS** | | **0.0000** | **1.74** | **SEPSECS** | **0.0041** | **1.61** | **MAP3K5** | | **0.0007** | **1.52** |
| **LOC644249** | **0.0006** | **2.17** | **ERV3-1** | | **0.0024** | **1.73** | **USP16** | **0.0004** | **1.61** | **CDK2** | | **0.0037** | **1.52** |
| **ANKRD20A5P** | **0.0022** | **2.16** | **BAZ2B** | | **0.0000** | **1.73** | **IPO8** | **0.0000** | **1.61** | **SRSF1** | | **0.0009** | **1.52** |
| **SNHG15** | **0.0002** | **2.14** | **ZBTB40** | | **0.0000** | **1.73** | **SNORA31** | **0.0014** | **1.60** | **ATP10D** | | **0.0018** | **1.52** |
| **EMC2** | **0.0012** | **2.12** | **ERCC5** | | **0.0007** | **1.73** | **EXOC1** | **0.0025** | **1.60** | **HERC2P4** | | **0.0002** | **1.52** |
| **CDK5RAP3** | **0.0020** | **2.12** | **MCM8** | | **0.0047** | **1.73** | **NAB1** | **0.0016** | **1.60** | **ZNF808** | | **0.0012** | **1.52** |
| **C6orf70** | **0.0003** | **2.10** | **C1orf27** | | **0.0007** | **1.73** | **PRKAB2** | **0.0005** | **1.60** | **HERC2P4** | | **0.0003** | **1.52** |
| **CD160** | **0.0022** | **2.09** | **SLC33A1** | | **0.0001** | **1.73** | **EDEM3** | **0.0029** | **1.60** | **STK35** | | **0.0018** | **1.52** |
| **MITD1** | **0.0003** | **2.08** | **PDZD4** | | **0.0020** | **1.72** | **CSRNP2** | **0.0002** | **1.60** | **SLC37A3** | | **0.0006** | **1.52** |
| **ZNF322** | **0.0002** | **2.08** | **ILF3-AS1** | | **0.0038** | **1.72** | **HIBCH** | **0.0022** | **1.60** | **SMC5** | | **0.0004** | **1.52** |
| **ANKZF1** | **0.0000** | **2.06** | **RFC4** | | **0.0003** | **1.72** | **YOD1** | **0.0031** | **1.60** | **MCM3AP** | | **0.0000** | **1.52** |
| **C5orf63** | **0.0009** | **2.06** | **ROCK1** | | **0.0013** | **1.72** | **AKTIP** | **0.0007** | **1.60** | **ARHGAP4** | | **0.0023** | **1.51** |
| **PIGT** | **0.0003** | **2.06** | **HERC2P2** | | **0.0000** | **1.72** | **EIF2B1** | **0.0012** | **1.59** | **FAM13B** | | **0.0010** | **1.51** |
| **CRYGS** | **0.0010** | **2.05** | **MFSD8** | | **0.0004** | **1.72** | **ACBD5** | **0.0027** | **1.59** | **ILKAP** | | **0.0000** | **1.51** |
| **PTGES3P1** | **0.0044** | **2.04** | **FTSJ3** | | **0.0036** | **1.72** | **KTN1** | **0.0005** | **1.59** | **HERC1** | | **0.0003** | **1.51** |
| **RAB28** | **0.0007** | **2.04** | **PLEKHA1** | | **0.0049** | **1.72** | **GMFB** | **0.0000** | **1.59** | **DDX27** | | **0.0000** | **1.51** |
| **ZNF780B** | **0.0009** | **2.03** | **ABCF3** | | **0.0014** | **1.72** | **CCAR1** | **0.0000** | **1.59** | **TMEM161B** | | **0.0006** | **1.51** |
| **GOLGA8A** | **0.0006** | **2.03** | **IFT20** | | **0.0008** | **1.71** | **ATR** | **0.0000** | **1.59** | **MC1R** | | **0.0025** | **1.51** |
| **ZNF558** | **0.0002** | **2.02** | **SSX2IP** | | **0.0005** | **1.71** | **UFL1** | **0.0003** | **1.59** | **SNUPN** | | **0.0039** | **1.51** |
| **GOLGA8B** | **0.0008** | **2.01** | **CLHC1** | | **0.0024** | **1.71** | **ZCRB1** | **0.0037** | **1.59** | **HEATR5B** | | **0.0015** | **1.51** |
| **GK5** | **0.0008** | **1.99** | **EFCAB4B** | | **0.0026** | **1.71** | **CDC37** | **0.0001** | **1.59** | **SRSF7** | | **0.0002** | **1.51** |
| **SPDL1** | **0.0004** | **1.99** | **TMEM50B** | | **0.0001** | **1.71** | **MRPS5** | **0.0016** | **1.58** | **ZNF692** | | **0.0032** | **1.51** |
| **SNORA21** | **0.0020** | **1.99** | **ZNF266** | | **0.0046** | **1.70** | **NADSYN1** | **0.0000** | **1.58** | **SMARCAD1** | | **0.0019** | **1.51** |
| **SP140L** | **0.0000** | **1.98** | **RAB27B** | | **0.0000** | **1.70** | **AGTPBP1** | **0.0003** | **1.58** | **KIAA1147** | | **0.0001** | **1.51** |
| **NAA40** | **0.0000** | **1.98** | **GLMN** | | **0.0007** | **1.70** | **BTN3A3** | **0.0001** | **1.58** | **ATG2B** | | **0.0001** | **1.51** |
| **SLFN13** | **0.0000** | **1.98** | **TRAPPC6B** | | **0.0023** | **1.70** | **EIF4G3** | **0.0027** | **1.58** | **KATNA1** | | **0.0005** | **1.51** |
| **ZNF585B** | **0.0024** | **1.98** | **IKBKAP** | | **0.0000** | **1.69** | **IKZF2** | **0.0002** | **1.58** | **PPOX** | | **0.0003** | **1.51** |
| **PPWD1** | **0.0000** | **1.97** | **JAKMIP2** | | **0.0047** | **1.69** | **GOLGA8I** | **0.0026** | **1.58** | **DDX42** | | **0.0006** | **1.51** |
| **CCDC66** | **0.0000** | **1.97** | **NHLRC2** | | **0.0021** | **1.69** | **ETFDH** | **0.0029** | **1.58** | **TAF1A** | | **0.0039** | **1.51** |
| **ARHGAP12** | **0.0000** | **1.96** | **ZDHHC6** | | **0.0000** | **1.69** | **MRPS31P5** | **0.0000** | **1.58** | **NPC1** | | **0.0020** | **1.51** |
| **NPFF** | **0.0003** | **1.96** | **ZFYVE16** | | **0.0001** | **1.68** | **UHRF2** | **0.0001** | **1.57** | **PDCD11** | | **0.0000** | **1.51** |
| **VAMP1** | **0.0001** | **1.96** | **ZDHHC21** | | **0.0019** | **1.68** | **NRD1** | **0.0000** | **1.57** | **NDUFS2** | | **0.0037** | **1.51** |
| **DZIP3** | **0.0022** | **1.95** | **LEO1** | | **0.0016** | **1.68** | **FARS2** | **0.0009** | **1.57** | **MAN2A2** | | **0.0002** | **1.51** |
| **LOC338799** | **0.0002** | **1.95** | **ALG13** | | **0.0007** | **1.68** | **SDHAP2** | **0.0024** | **1.57** | **MSTO1** | | **0.0045** | **1.51** |
| **ANAPC4** | **0.0000** | **1.95** | **PTAR1** | | **0.0003** | **1.68** | **LAIR1** | **0.0016** | **1.57** | **ZNF248** | | **0.0007** | **1.50** |
| **MAPKAPK5-AS1** | **0.0001** | **1.95** | **SLC35A3** | | **0.0007** | **1.68** | **INSIG2** | **0.0017** | **1.57** | **DNAJB11** | | **0.0022** | **1.50** |
| **GAS5** | **0.0010** | **1.95** | **PWP2** | | **0.0001** | **1.68** | **NPIPA5** | **0.0016** | **1.57** | **PLEKHG3** | | **0.0018** | **1.50** |
| **CAPRIN2** | **0.0000** | **1.94** | **NOM1** | | **0.0000** | **1.67** | **ZNF70** | **0.0029** | **1.56** | **ZMYM6** | | **0.0008** | **1.50** |
| **KLHDC1** | **0.0000** | **1.93** | **VAMP4** | | **0.0042** | **1.67** | **USP47** | **0.0004** | **1.56** | **DBR1** | | **0.0020** | **1.50** |
| **ARGLU1** | **0.0001** | **1.93** | **ZRANB2** | | **0.0016** | **1.67** | **FAM114A2** | **0.0015** | **1.56** | **SMURF2** | | **0.0003** | **1.50** |
| **CEP95** | **0.0000** | **1.93** | **PMPCB** | | **0.0014** | **1.67** | **TTC13** | **0.0001** | **1.56** | **PSTPIP1** | | **0.0019** | **1.50** |
| **ZNF224** | **0.0003** | **1.93** | **ANKRD36C** | | **0.0015** | **1.67** | **KIAA0368** | **0.0004** | **1.56** | **INPP4A** | | **0.0009** | **1.50** |
| **OTTHUMG00000152788** | **0.0000** | **1.93** | **NS3BP** | | **0.0042** | **1.67** | **GOLGA1** | **0.0010** | **1.56** | **NXF1** | | **0.0013** | **1.50** |
| **GBP3** | **0.0005** | **1.92** | **LYG1** | | **0.0011** | **1.67** | **LPAL2** | **0.0015** | **1.56** | **DHX38** | | **0.0035** | **1.50** |
| **EXOSC8** | **0.0000** | **1.92** | **GPD1L** | | **0.0018** | **1.67** | **NDUFV1** | **0.0040** | **1.56** | **ERBB2IP** | | **0.0011** | **1.50** |
| **MGME1** | **0.0007** | **1.92** | **ARID4A** | | **0.0000** | **1.67** | **CD226** | **0.0026** | **1.56** | **SDHAP1** | | **0.0002** | **1.50** |
| **YTHDC2** | **0.0000** | **1.91** | **SYNE1** | | **0.0001** | **1.66** | **NAA35** | **0.0000** | **1.56** | **VPS8** | | **0.0001** | **1.50** |
| **SNORD38A** | **0.0026** | **1.90** | **DHX29** | | **0.0008** | **1.66** | **LINC00674** | **0.0015** | **1.56** | **ZKSCAN8** | | **0.0042** | **1.50** |
| **RARS** | **0.0001** | **1.90** | **CCDC104** | | **0.0008** | **1.66** | **PRMT10** | **0.0037** | **1.56** |  | |  |  |

**Additional file 1: Table S1. Genes up regulated in SVR vs. NR**

**Legend**: Genes are derived from Student’s t test (p value < 0.005; Fold Change (FC) > 1.5) between HCV responding (SVR) vs. non responding (NR) patients to Interferon-α + Ribavirin treatment. Genes are ordered based on descending parametric FC. GENE name refers to official gene symbol (http://www.ncbi.nlm.nih.gov/gene/).
