# Supplementary material for: Inherent transcriptional signatures of NK cells are associated with response to IFNα + rivabirin therapy in patients with Hepatitis C Virus
Source: J Transl Med. 2015 Mar 1;13:77. doi: 10.1186/s12967-015-0428-x (PMC4353456; doi:10.1186/s12967-015-0428-x)
Supplement: Additional file 2: Table S2. — Student’s T test SVR vs. NR (p < 0.005; FC < −1.5). List of 60 transcripts down regulated in SVR patients. Transcripts are ordered based on descending Fold Change (FC). [file 12967_2015_428_MOESM2_ESM.docx]

| **Gene** | **P value** | | **FC** | **Gene** | **P value** | **FC** | **Gene** | **P value** | **FC** |
| --- | --- | --- | --- | --- | --- | --- | --- | --- | --- |
| **HLA-DQA1** | **3E-04** | **-5.40** | | **CD1C** | **2E-03** | **-1.97** | **TCF7** | **5E-03** | **-1.59** |
| **IGKV3D-11** | **3E-03** | **-4.50** | | **LY6E** | **2E-03** | **-1.91** | **DNAJC15** | **4E-03** | **-1.59** |
| **IGHV4-61** | **4E-03** | **-4.27** | | **MYC** | **2E-03** | **-1.86** | **NINJ1** | **2E-03** | **-1.58** |
| **EPC1** | **4E-03** | **-3.20** | | **RNA5SP339** | **5E-03** | **-1.84** | **PSENEN** | **4E-03** | **-1.58** |
| **RNY3P3** | **8E-05** | **-3.13** | | **RNA5SP456** | **2E-03** | **-1.77** | **ASF1B** | **4E-03** | **-1.57** |
| **CD79A** | **3E-03** | **-2.86** | | **SPIB** | **3E-03** | **-1.77** | **RNA5SP497** | **3E-04** | **-1.57** |
| **HLA-DQB1** | **7E-04** | **-2.82** | | **POU2AF1** | **3E-03** | **-1.77** | **CLEC17A** | **3E-04** | **-1.56** |
| **BLNK** | **2E-03** | **-2.47** | | **XCL1** | **2E-03** | **-1.76** | **ITM2C** | **3E-03** | **-1.56** |
| **VPREB3** | **2E-03** | **-2.39** | | **RNA5SP178** | **3E-03** | **-1.75** | **AMPD3** | **3E-04** | **-1.56** |
| **FCRL1** | **5E-03** | **-2.36** | | **P2RX5** | **5E-03** | **-1.74** | **CYP4A22** | **2E-03** | **-1.55** |
| **HLA-DQA2** | **7E-04** | **-2.26** | | **TLR7** | **1E-03** | **-1.73** | **C8orf74** | **3E-04** | **-1.55** |
| **PAX5** | **2E-03** | **-2.25** | | **HLA-DQB2** | **9E-04** | **-1.72** | **NOTUM** | **2E-04** | **-1.55** |
| **GNG7** | **5E-03** | **-2.22** | | **FLJ20464** | **2E-03** | **-1.68** | **LTB** | **1E-03** | **-1.54** |
| **ADAM19** | **2E-03** | **-2.22** | | **KRTAP10-2** | **8E-06** | **-1.65** | **BIRC3** | **3E-03** | **-1.54** |
| **SNORA70E** | **2E-04** | **-2.16** | | **PGLS** | **6E-04** | **-1.64** | **OR6N2** | **7E-04** | **-1.53** |
| **EBF1** | **1E-03** | **-2.10** | | **TRAPPC6A** | **1E-03** | **-1.63** | **OTTHUMG00000156757** | **2E-03** | **-1.53** |
| **CD74** | **3E-04** | **-2.07** | | **EIF4EBP1** | **1E-03** | **-1.63** | **VN1R17P** | **3E-03** | **-1.52** |
| **LOC100134663** | **5E-04** | **-2.01** | | **MIF** | **2E-03** | **-1.62** | **RNA5SP518** | **8E-04** | **-1.51** |
| **GTSF1** | **2E-03** | **-2.00** | | **LOC652276** | **1E-03** | **-1.61** | **SSTR5-AS1** | **5E-03** | **-1.51** |
| **PPDPF** | **2E-04** | **-1.98** | | **GZMM** | **5E-04** | **-1.60** | **FAM71E2** | **6E-05** | **-1.51** |

**Additional file 2: Table S2. Gene down regulated in SVR vs. NR**

**Legend**: Genes are derived from Student’s t test (p value < 0.005; Fold Change (FC) < -1.5) between HCV responding (SVR) vs. non responding (NR) patients to Interferon-α + Ribavirin treatment. Genes are ordered based on descending parametric FC. GENE ID refers to official gene symbol (http://www.ncbi.nlm.nih.gov/gene/).
