# Supplementary material for: Inherent transcriptional signatures of NK cells are associated with response to IFNα + rivabirin therapy in patients with Hepatitis C Virus
Source: J Transl Med. 2015 Mar 1;13:77. doi: 10.1186/s12967-015-0428-x (PMC4353456; doi:10.1186/s12967-015-0428-x)
Supplement: Additional file 3: Table S3. — Student’s T test CV-HCV vs. HD (p < 0.005; FC > 1.5 or Fc < −1.5). List of 165 genes up-regulated and down regulated in CV-HCV compared to HD. Transcripts are ordered based on descending Fold Change (FC). [file 12967_2015_428_MOESM3_ESM.docx]

| **Gene** | **P value** | **FC** | **Gene** | **P value** | **FC** | **Gene** | **P value** | **FC** |
| --- | --- | --- | --- | --- | --- | --- | --- | --- |
| TP53TG5 | 0.0001 | 2.95 | HSPA13 | 0.0001 | -1.53 | RNU6V | 0.0003 | -1.68 |
| TP53TG5 | 0.0001 | 2.95 | RLIM | 0.0001 | -1.53 | RBMS1P1 | 0.0000 | -1.68 |
| RSAD2 | 0.0007 | 2.80 | SLC38A2 | 0.0000 | -1.54 | PAPD5 | 0.0000 | -1.69 |
| SCARNA7 | 0.0000 | 2.59 | RIPK2 | 0.0002 | -1.54 | CHD1 | 0.0003 | -1.69 |
| OR4G3P | 0.0039 | 2.38 | IL6ST | 0.0009 | -1.54 | EIF4A2 | 0.0012 | -1.71 |
| OR4G3P | 0.0039 | 2.38 | SNORD82 | 0.0047 | -1.54 | RGPD8 | 0.0000 | -1.72 |
| GSTM2 | 0.0003 | 2.33 | GADD45A | 0.0021 | -1.54 | LY6G5B | 0.0005 | -1.72 |
| IGKV1D-43 | 0.0034 | 2.22 | TUBA4A | 0.0015 | -1.54 | TAF1D | 0.0014 | -1.72 |
| OR11H12 | 0.0018 | 2.08 | CHUK | 0.0003 | -1.54 | TM6SF1 | 0.0001 | -1.72 |
| HIST1H2BM | 0.0001 | 2.05 | OTTHUMG00000157481 | 0.0008 | -1.54 | ZNF14 | 0.0045 | -1.73 |
| BTLA | 0.0046 | 2.02 | SPTY2D1 | 0.0000 | -1.54 | SLED1 | 0.0042 | -1.74 |
| FCRL1 | 0.0029 | 2.00 | USPL1 | 0.0022 | -1.55 | AMZ2P1 | 0.0033 | -1.74 |
| RNU1-11P | 0.0004 | 1.91 | ING2 | 0.0002 | -1.55 | RNF138 | 0.0000 | -1.74 |
| LOC100134663 | 0.0001 | 1.90 | LOC100130428 | 0.0041 | -1.55 | RGPD4 | 0.0000 | -1.75 |
| IFIT1 | 0.0023 | 1.87 | ADIPOR1 | 0.0003 | -1.56 | CD160 | 0.0020 | -1.75 |
| SNORA46 | 0.0002 | 1.87 | ACSL3 | 0.0033 | -1.56 | SERTAD2 | 0.0003 | -1.76 |
| HIST1H2BO | 0.0011 | 1.77 | BTG2 | 0.0008 | -1.56 | LINC00528 | 0.0024 | -1.76 |
| HIST1H1D | 0.0001 | 1.72 | DDX20 | 0.0001 | -1.56 | CD83 | 0.0029 | -1.78 |
| LOC100128751 | 0.0003 | 1.71 | SLC25A33 | 0.0011 | -1.56 | RGPD1 | 0.0000 | -1.78 |
| XAF1 | 0.0031 | 1.68 | KLF11 | 0.0005 | -1.57 | OTTHUMG00000166675 | 0.0006 | -1.78 |
| CETN3 | 0.0043 | 1.65 | KBTBD2 | 0.0000 | -1.57 | RGS2 | 0.0044 | -1.78 |
| EBF1 | 0.0028 | 1.65 | SLC35A3 | 0.0002 | -1.57 | PIM3 | 0.0000 | -1.78 |
| TMA16 | 0.0016 | 1.62 | SNORD48 | 0.0042 | -1.57 | EIF4A1 | 0.0035 | -1.79 |
| GIMAP6 | 0.0027 | 1.62 | ZFP36 | 0.0014 | -1.57 | FAM105A | 0.0000 | -1.79 |
| HIST1H4D | 0.0004 | 1.60 | AP3M2 | 0.0026 | -1.58 | SCARNA9L | 0.0007 | -1.80 |
| GIMAP8 | 0.0008 | 1.60 | JHDM1D | 0.0000 | -1.58 | NR1D2 | 0.0000 | -1.80 |
| BYSL | 0.0006 | 1.58 | MAPKAPK5-AS1 | 0.0003 | -1.58 | RNU4-9P | 0.0033 | -1.81 |
| ERVH-4 | 0.0007 | 1.56 | SNORA70C | 0.0000 | -1.58 | IER3 | 0.0004 | -1.82 |
| GIMAP5 | 0.0034 | 1.54 | NFKB1 | 0.0002 | -1.59 | SNORA45 | 0.0000 | -1.82 |
| TPTE2 | 0.0048 | 1.54 | CDHR1 | 0.0005 | -1.59 | MAFG | 0.0001 | -1.82 |
| P2RX5 | 0.0040 | 1.53 | C3orf58 | 0.0001 | -1.59 | SNORD63 | 0.0037 | -1.83 |
| GID8 | 0.0020 | -1.50 | SNORA27 | 0.0028 | -1.60 | RGPD5 | 0.0000 | -1.84 |
| TGFB1 | 0.0000 | -1.50 | LY6G5B | 0.0013 | -1.60 | SNORD38A | 0.0004 | -1.85 |
| MPC1 | 0.0000 | -1.50 | SKIL | 0.0009 | -1.60 | LDHA | 0.0000 | -1.85 |
| TIPARP | 0.0005 | -1.50 | TGFBRAP1 | 0.0002 | -1.61 | CPEB2 | 0.0004 | -1.86 |
| EOGT | 0.0001 | -1.50 | CRY1 | 0.0006 | -1.61 | ELL2 | 0.0019 | -1.88 |
| GAS5 | 0.0046 | -1.50 | PRKRIR | 0.0002 | -1.61 | BRMS1L | 0.0000 | -1.92 |
| ZC3H12A | 0.0006 | -1.50 | GNAQ | 0.0043 | -1.61 | RNU6-69P | 0.0000 | -1.98 |
| HNRNPA1 | 0.0001 | -1.50 | DCTN4 | 0.0001 | -1.61 | LRRFIP1 | 0.0034 | -1.98 |
| PGK1 | 0.0011 | -1.50 | DBF4 | 0.0015 | -1.62 | TNFAIP3 | 0.0012 | -1.98 |
| MAML2 | 0.0000 | -1.51 | TANC2 | 0.0000 | -1.63 | GZF1 | 0.0000 | -2.04 |
| ZNF267 | 0.0006 | -1.51 | DENND1B | 0.0002 | -1.63 | OCR1 | 0.0002 | -2.06 |
| SLC38A1 | 0.0002 | -1.51 | SNORD57 | 0.0004 | -1.63 | ZRANB2 | 0.0002 | -2.09 |
| FAM102B | 0.0000 | -1.51 | LOC729603 | 0.0004 | -1.63 | ELL2P1 | 0.0022 | -2.15 |
| CDKN2AIP | 0.0022 | -1.51 | OSER1 | 0.0000 | -1.63 | SIK1 | 0.0021 | -2.17 |
| FAM105B | 0.0001 | -1.51 | HIAT1 | 0.0001 | -1.63 | SLC7A5 | 0.0019 | -2.27 |
| RPL13 | 0.0044 | -1.52 | FBXO33 | 0.0000 | -1.64 | SMAD7 | 0.0006 | -2.31 |
| RABGGTB | 0.0003 | -1.52 | ZDHHC7 | 0.0001 | -1.64 | SCML1 | 0.0025 | -2.35 |
| NCOA1 | 0.0000 | -1.52 | SNHG17 | 0.0000 | -1.64 | GAFA2 | 0.0002 | -2.35 |
| AAK1 | 0.0000 | -1.52 | TAF9B | 0.0004 | -1.64 | PIGT | 0.0000 | -2.44 |
| YOD1 | 0.0009 | -1.52 | TAF9B | 0.0004 | -1.64 | NR4A2 | 0.0010 | -2.58 |
| DNTTIP2 | 0.0001 | -1.53 | RLF | 0.0001 | -1.66 | RGS1 | 0.0017 | -2.77 |
| CCDC109B | 0.0028 | -1.53 | PDPR | 0.0001 | -1.66 | TUBB1 | 0.0044 | -3.17 |
| SRSF2 | 0.0001 | -1.53 | DDX18P1 | 0.0013 | -1.67 |  |  |  |
| CHKA | 0.0009 | -1.53 | AHR | 0.0016 | -1.67 |  |  |  |
| PPT1 | 0.0029 | -1.53 | WDR47 | 0.0016 | -1.68 |  |  |  |

**Additional file 3: Table S3. Genes up and down regulated in CV-HCV vs. HD**

**Legend**: Genes are derived from Student’s t test (p value < 0.005; Fold Change (FC) > 1.5 or < -1.5) between HCV responding (SVR) + non responding (NR) patients (together denominated CV-HCV) vs. healthy individuals (HD). Genes are ordered based on descending parametric FC. Genes with positive FC are up regulated in CV patients. Genes with negative FC are down regulated in CV patients. GENE name refers to official gene symbol (http://www.ncbi.nlm.nih.gov/gene/).
