# Supplementary material for: Inherent transcriptional signatures of NK cells are associated with response to IFNα + rivabirin therapy in patients with Hepatitis C Virus
Source: J Transl Med. 2015 Mar 1;13:77. doi: 10.1186/s12967-015-0428-x (PMC4353456; doi:10.1186/s12967-015-0428-x)
Supplement: Additional file 4: Table S4. — Screening of IL28B rs12979860 polymorphism in the training and validating groups of HCV patients. [file 12967_2015_428_MOESM4_ESM.docx]

| **Sample ID** | | **Group legend** | **Treatment naïve** | **HCV Treatment Response** | **IL28B**  **rs12979860** |
| --- | --- | --- | --- | --- | --- |
| GEp4 | Training group | | Yes | NR | CT |
| GEp5 | Training group | | Yes | NR | TT |
| GEp53 | Training group | | Yes | NR | CC |
| GEp54 | Training group | | Yes | NR | CC |
| GEp15 | Training group | | Yes | NR | CC |
| GEp8 | Training group | | Yes | SVR | CT |
| GEp10 | Training group | | Yes | SVR | CC |
| GEp14 | Training group | | Yes | SVR | CC |
| GEp6 | Training group | | Yes | SVR | CT |
| GEp1 | Validating group | | Yes | SVR | CC |
| GEp2 | Validating group | | Yes | SVR | CT |
| GEp7 | Validating group | | Yes | SVR | TT |
| GEp9 | Validating group | | Yes | SVR | CT |
| GEp12 | Validating group | | Yes | SVR | CC |
| GEp17 | Validating group | | Yes | NR | CT |
| GEp18 | Validating group | | Yes | NR | CC |
| GEp19 | Validating group | | Yes | NR | CC |
| GEp20 | Validating group | | Yes | NR | CT |
| GEp21 | Validating group | | Yes | NR | CC |

**Additional file 4: Table S4. Screening of IL28B *rs12979860* polymorphism**

Legend: IL28B *rs12979860* polymorphism screening was performed by RT-PCR using DNA extracted from PBMCs of both the training and validating group of HCV-1 patients. These results showed that CC homozygosis was not significantly associated with SVR in our series of patients (F test SVR vs NR p =0.99)
